# Supplementary figures and images for: Expression patterns and the prognostic value of the EMILIN/Multimerin family members in low-grade glioma
Source: PeerJ. 2020 Mar 2;8:e8696. doi: 10.7717/peerj.8696 (PMC7058105; doi:10.7717/peerj.8696)

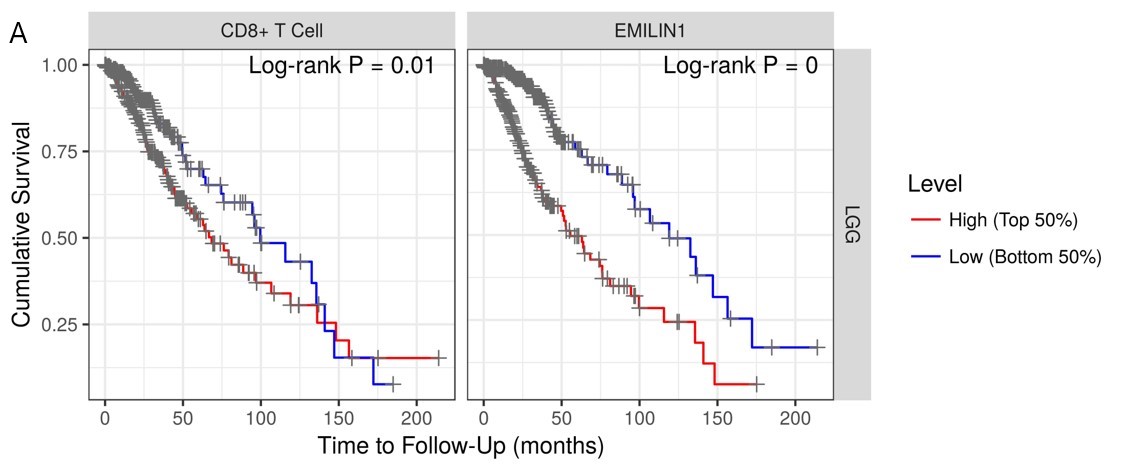

Supplement: Supplemental Information 1 [file peerj-08-8696-s001.zip › Raw data/TIMER survivel/═╝╞1⁄41.jpg]

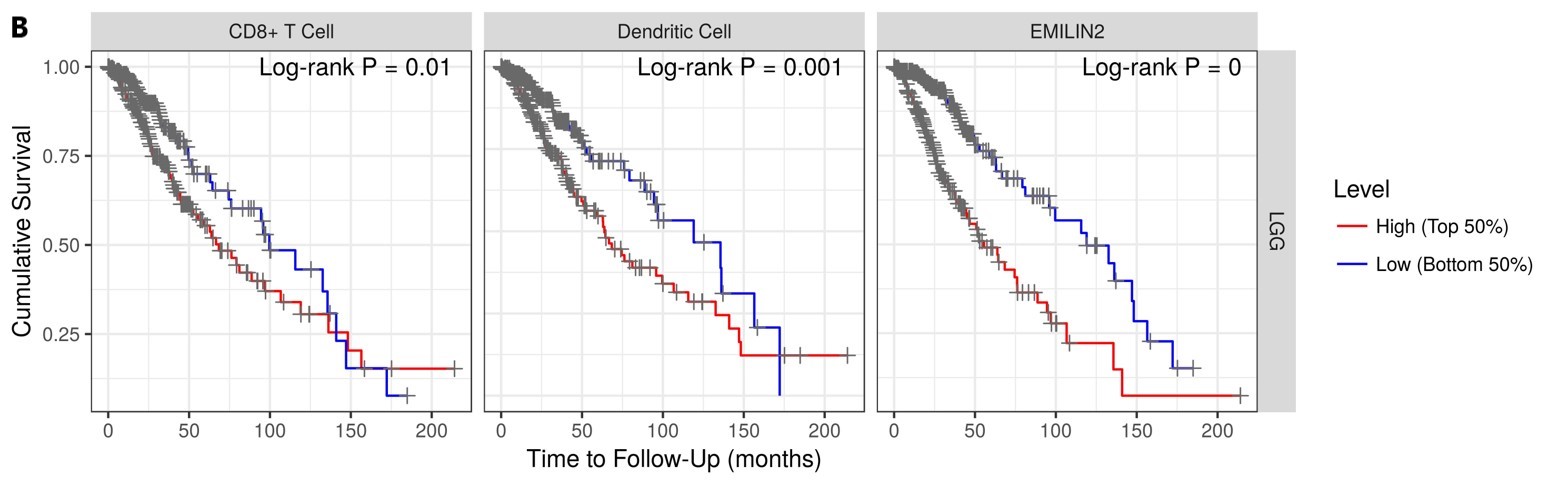

Supplement: Supplemental Information 1 [file peerj-08-8696-s001.zip › Raw data/TIMER survivel/═╝╞1⁄42.jpg]

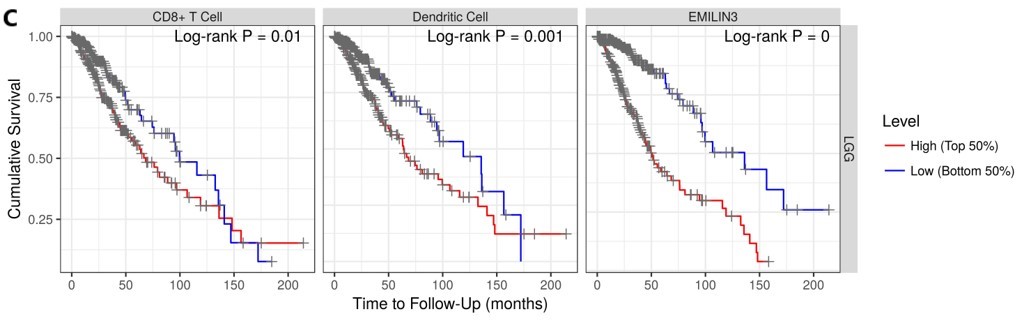

Supplement: Supplemental Information 1 [file peerj-08-8696-s001.zip › Raw data/TIMER survivel/═╝╞1⁄43.jpg]

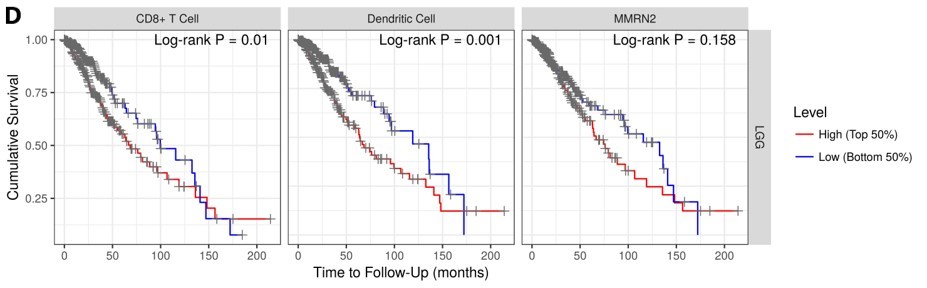

Supplement: Supplemental Information 1 [file peerj-08-8696-s001.zip › Raw data/TIMER survivel/═╝╞1⁄44.jpg]

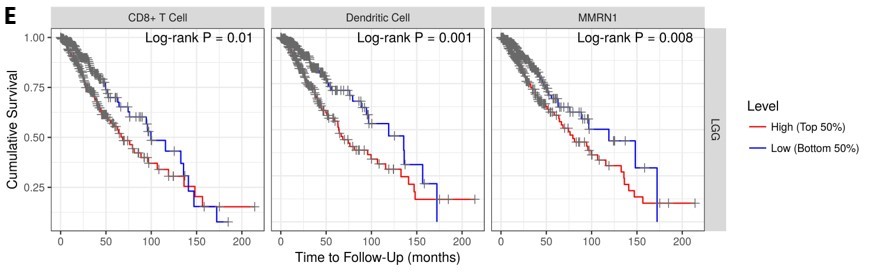

Supplement: Supplemental Information 1 [file peerj-08-8696-s001.zip › Raw data/TIMER survivel/═╝╞1⁄45.jpg]

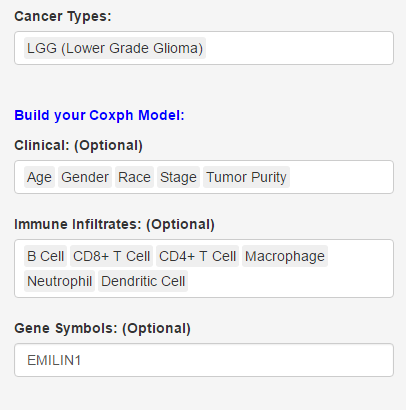

Supplement: Supplemental Information 1 [file peerj-08-8696-s001.zip › Raw data/TIMER survivel/╦╤╣╖╜╪═╝19─Ω05╘┬31╚╒1947_1.png]

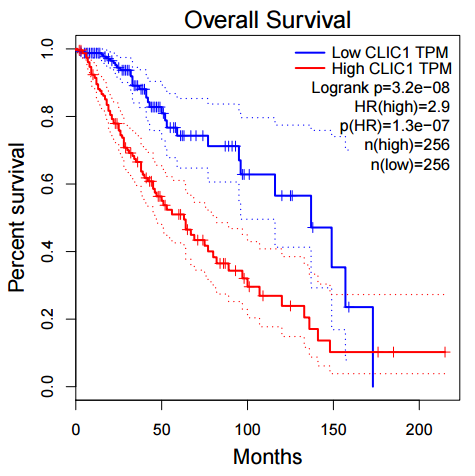

Supplement: Supplemental Information 1 [file peerj-08-8696-s001.zip › Raw data/co-expressed genes/CEPIA co survival/CLIC1.png]

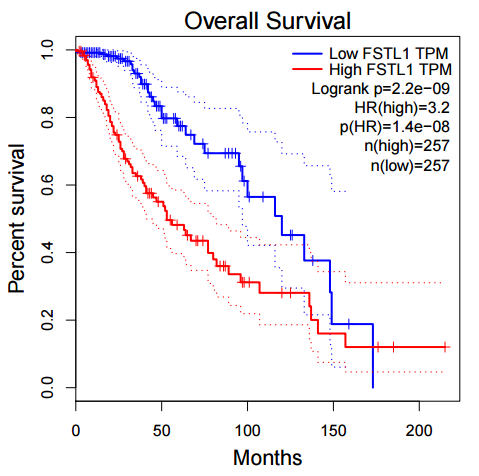

Supplement: Supplemental Information 1 [file peerj-08-8696-s001.zip › Raw data/co-expressed genes/CEPIA co survival/FSTL1.png]

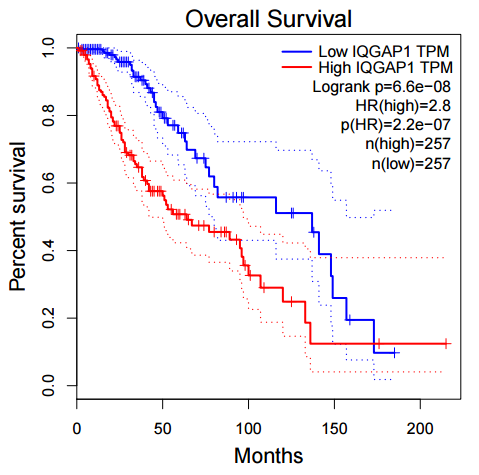

Supplement: Supplemental Information 1 [file peerj-08-8696-s001.zip › Raw data/co-expressed genes/CEPIA co survival/IQGAP1.png]

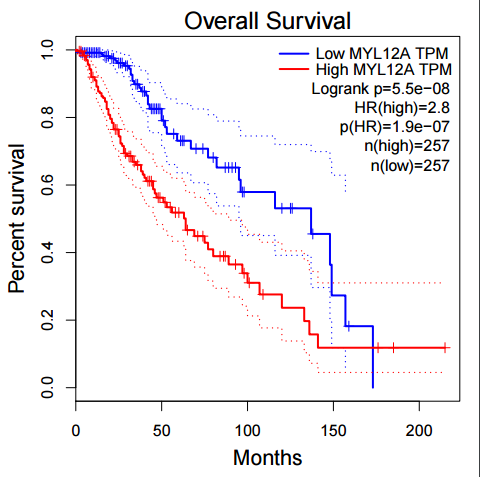

Supplement: Supplemental Information 1 [file peerj-08-8696-s001.zip › Raw data/co-expressed genes/CEPIA co survival/MYL12A.png]

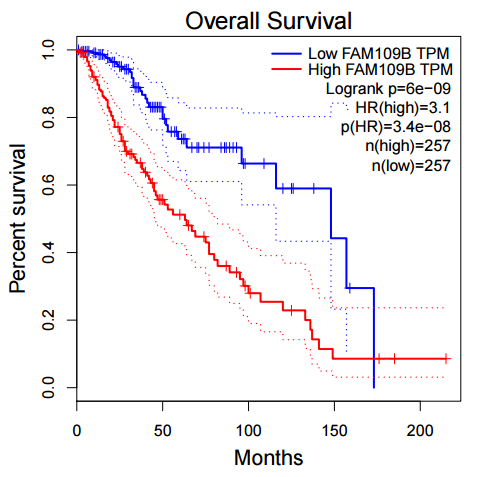

Supplement: Supplemental Information 1 [file peerj-08-8696-s001.zip › Raw data/co-expressed genes/CEPIA co survival/PHETA2 previous name is 109B.png]

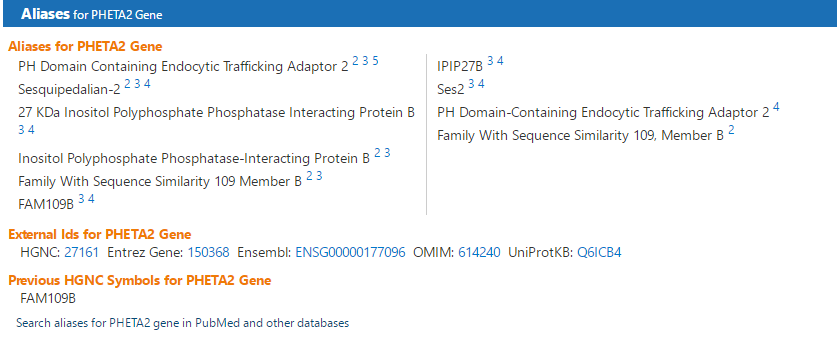

Supplement: Supplemental Information 1 [file peerj-08-8696-s001.zip › Raw data/co-expressed genes/CEPIA co survival/PHETA2 previous name is 109B.╓ñ├≈png.png]

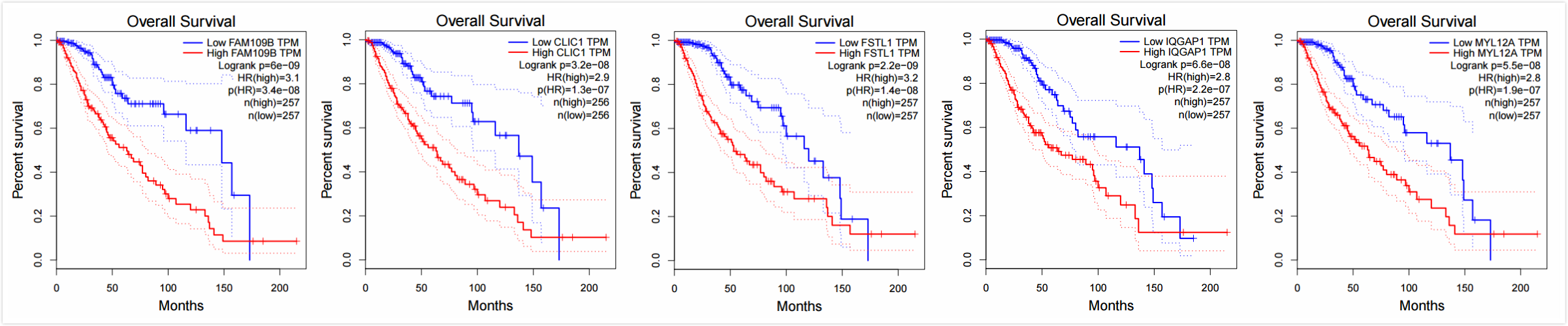

Supplement: Supplemental Information 1 [file peerj-08-8696-s001.zip › Raw data/co-expressed genes/CEPIA co survival/survival ╝⌠╟╨║≤.png]

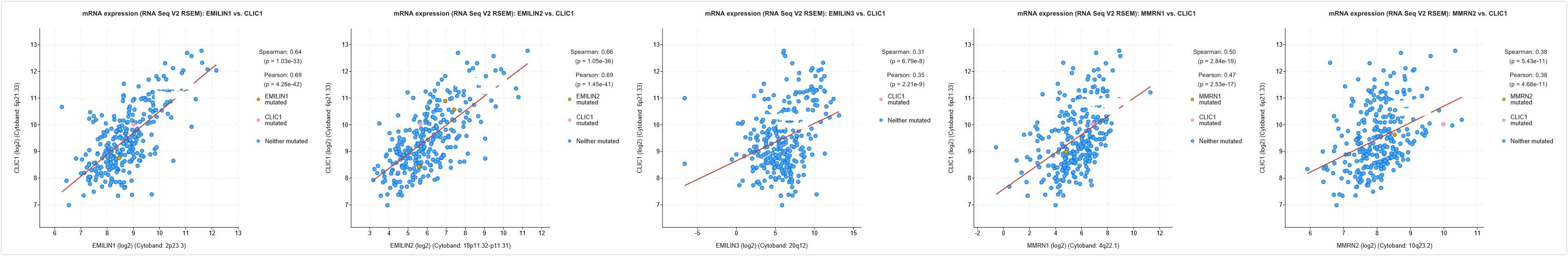

Supplement: Supplemental Information 1 [file peerj-08-8696-s001.zip › Raw data/co-expressed genes/CLIC1 ╝⌠╟╨/CLCL1 ╝⌠╟╨.jpg]

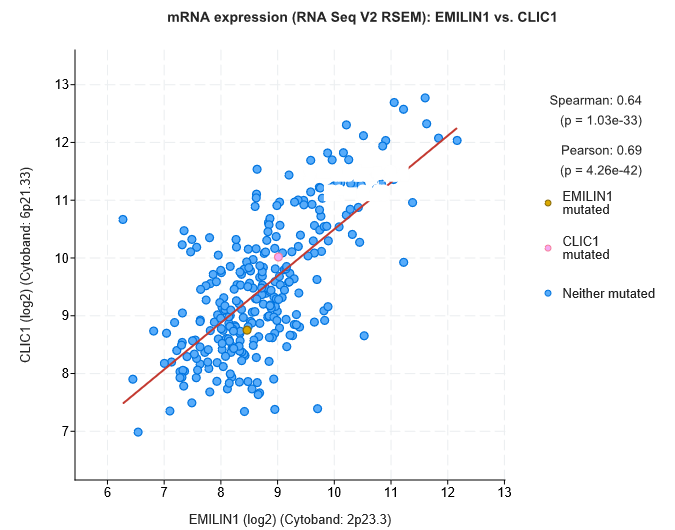

Supplement: Supplemental Information 1 [file peerj-08-8696-s001.zip › Raw data/co-expressed genes/CLIC1 ╝⌠╟╨/CLIC1%20EMILIN1.png]

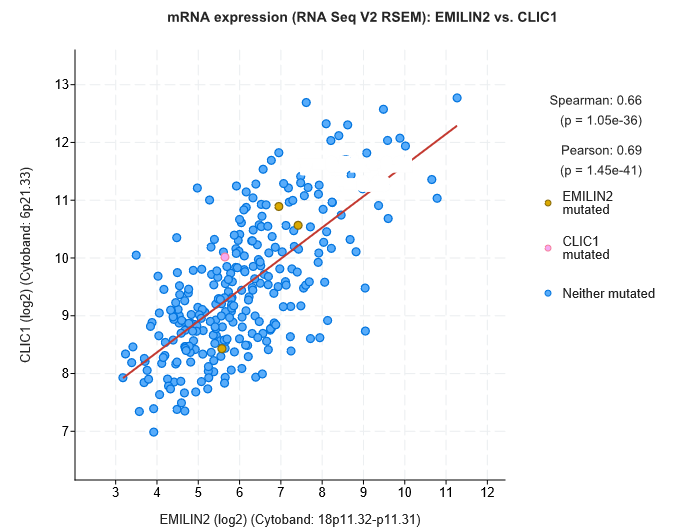

Supplement: Supplemental Information 1 [file peerj-08-8696-s001.zip › Raw data/co-expressed genes/CLIC1 ╝⌠╟╨/CLIC1%20EMILIN2.png]

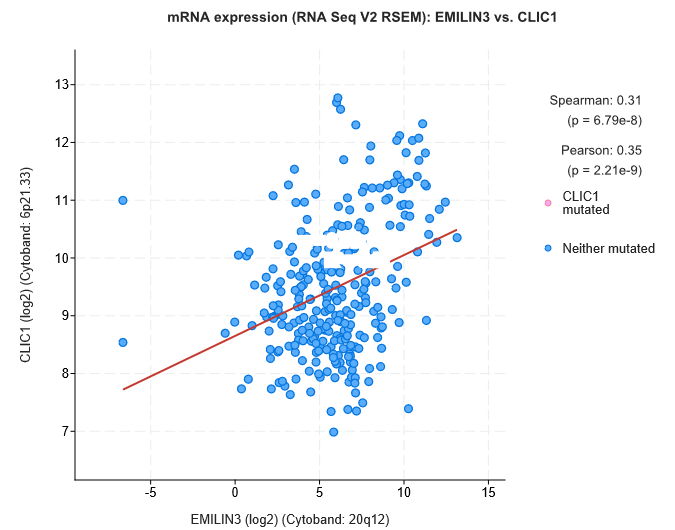

Supplement: Supplemental Information 1 [file peerj-08-8696-s001.zip › Raw data/co-expressed genes/CLIC1 ╝⌠╟╨/CLIC1%20EMILIN3.png]

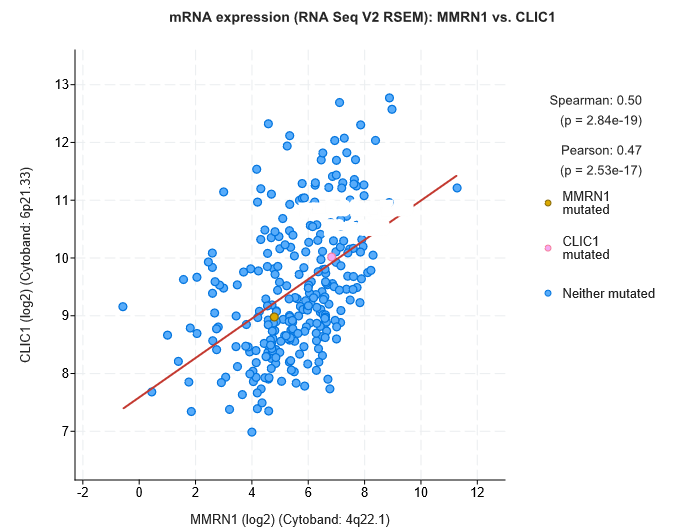

Supplement: Supplemental Information 1 [file peerj-08-8696-s001.zip › Raw data/co-expressed genes/CLIC1 ╝⌠╟╨/CLIC1%20MMRN1.png]

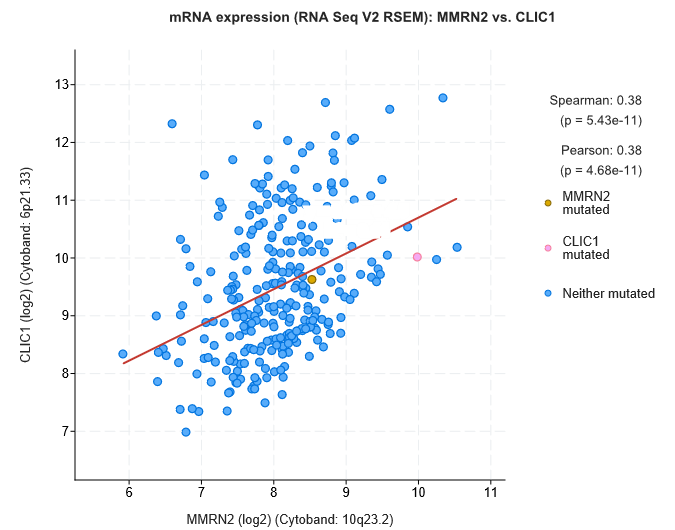

Supplement: Supplemental Information 1 [file peerj-08-8696-s001.zip › Raw data/co-expressed genes/CLIC1 ╝⌠╟╨/CLIC1%20MMRN2.png]

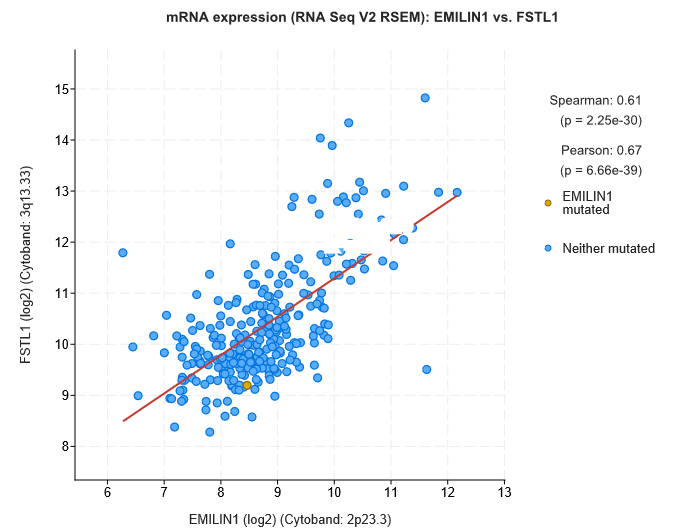

Supplement: Supplemental Information 1 [file peerj-08-8696-s001.zip › Raw data/co-expressed genes/FSTL1 ╝⌠╟╨/FSTL1%20EMILIN1.png]

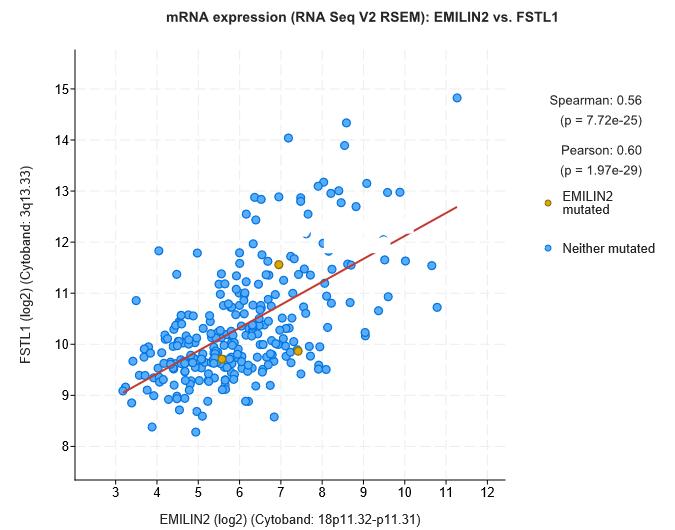

Supplement: Supplemental Information 1 [file peerj-08-8696-s001.zip › Raw data/co-expressed genes/FSTL1 ╝⌠╟╨/FSTL1%20EMILIN2.png]

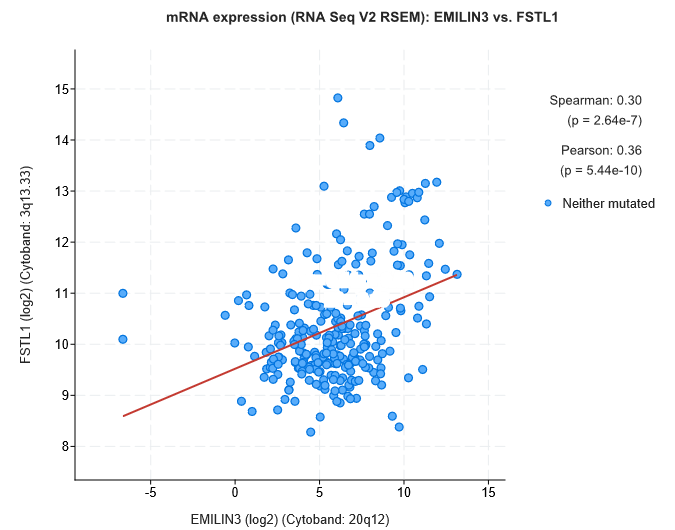

Supplement: Supplemental Information 1 [file peerj-08-8696-s001.zip › Raw data/co-expressed genes/FSTL1 ╝⌠╟╨/FSTL1%20EMILIN3.png]

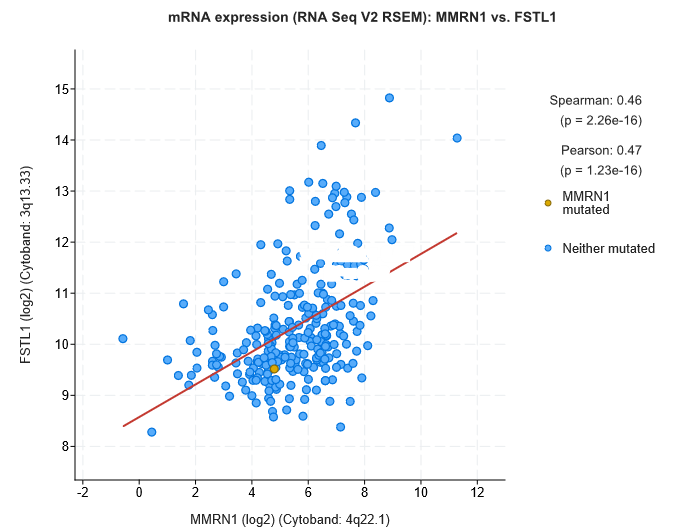

Supplement: Supplemental Information 1 [file peerj-08-8696-s001.zip › Raw data/co-expressed genes/FSTL1 ╝⌠╟╨/FSTL1%20MMRN1.png]

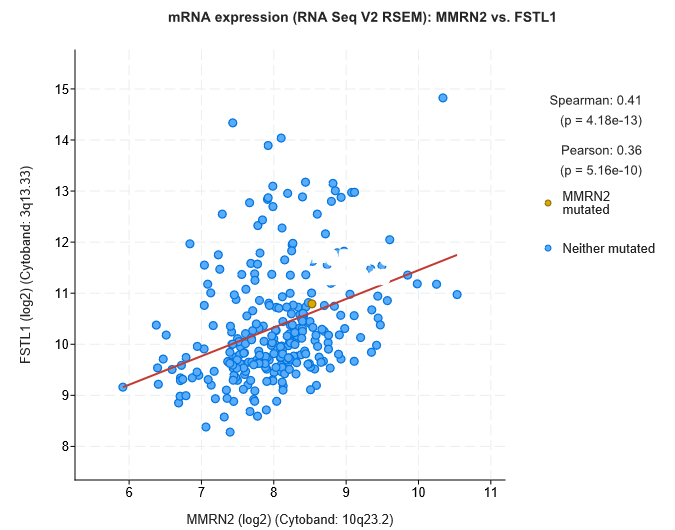

Supplement: Supplemental Information 1 [file peerj-08-8696-s001.zip › Raw data/co-expressed genes/FSTL1 ╝⌠╟╨/FSTL1%20MMRN2.png]

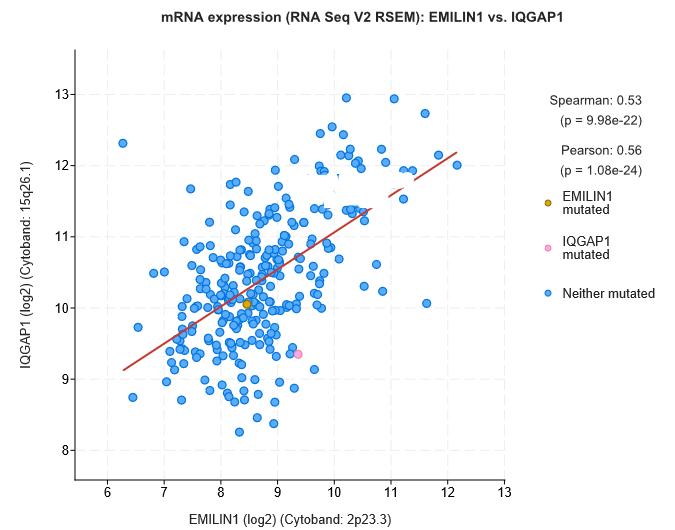

Supplement: Supplemental Information 1 [file peerj-08-8696-s001.zip › Raw data/co-expressed genes/IQGAP╝⌠╟╨/IQGAP1%20EMILIN1.png]

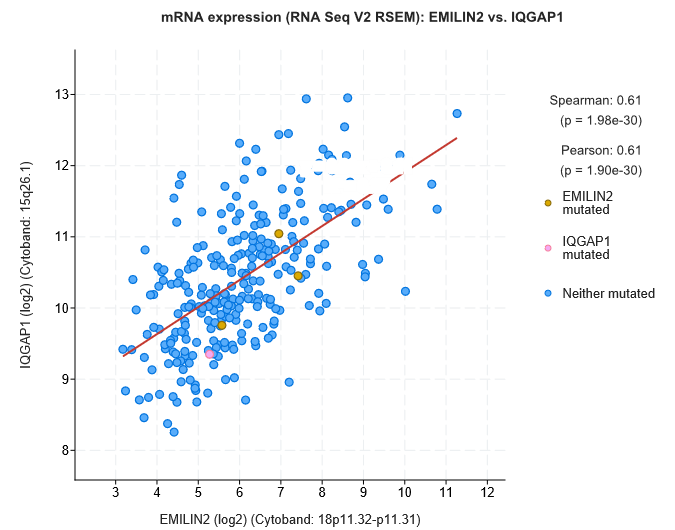

Supplement: Supplemental Information 1 [file peerj-08-8696-s001.zip › Raw data/co-expressed genes/IQGAP╝⌠╟╨/IQGAP1%20EMILIN2.png]

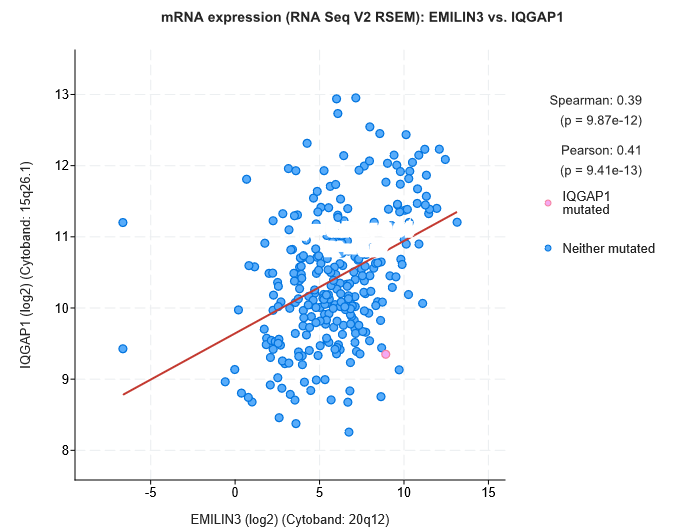

Supplement: Supplemental Information 1 [file peerj-08-8696-s001.zip › Raw data/co-expressed genes/IQGAP╝⌠╟╨/IQGAP1%20EMILIN3.png]

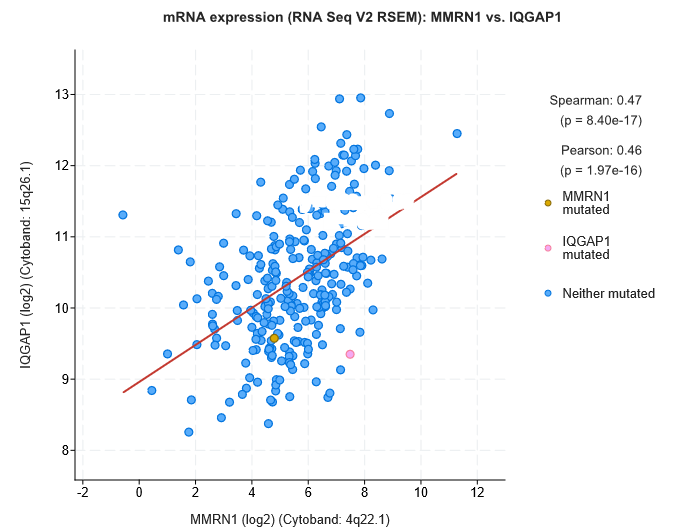

Supplement: Supplemental Information 1 [file peerj-08-8696-s001.zip › Raw data/co-expressed genes/IQGAP╝⌠╟╨/IQGAP1%20MMRN1.png]

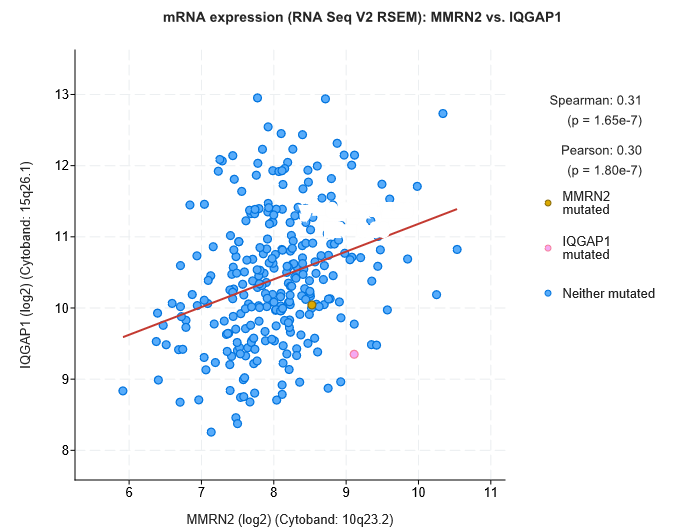

Supplement: Supplemental Information 1 [file peerj-08-8696-s001.zip › Raw data/co-expressed genes/IQGAP╝⌠╟╨/IQGAP1%20MMRN2.png]

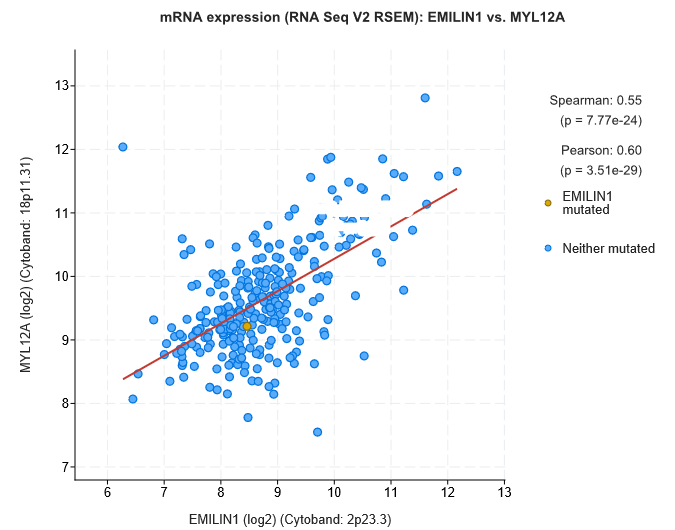

Supplement: Supplemental Information 1 [file peerj-08-8696-s001.zip › Raw data/co-expressed genes/MYL12A ╝⌠╟╨/MYL12A%20EMILIN1.png]

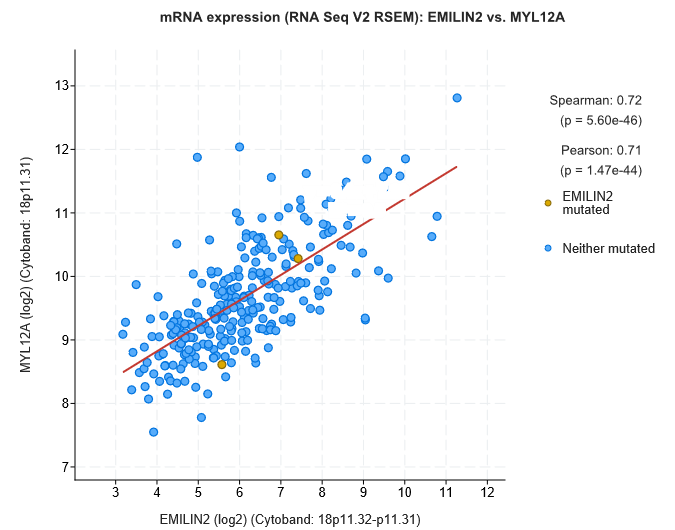

Supplement: Supplemental Information 1 [file peerj-08-8696-s001.zip › Raw data/co-expressed genes/MYL12A ╝⌠╟╨/MYL12A%20EMILIN2.png]

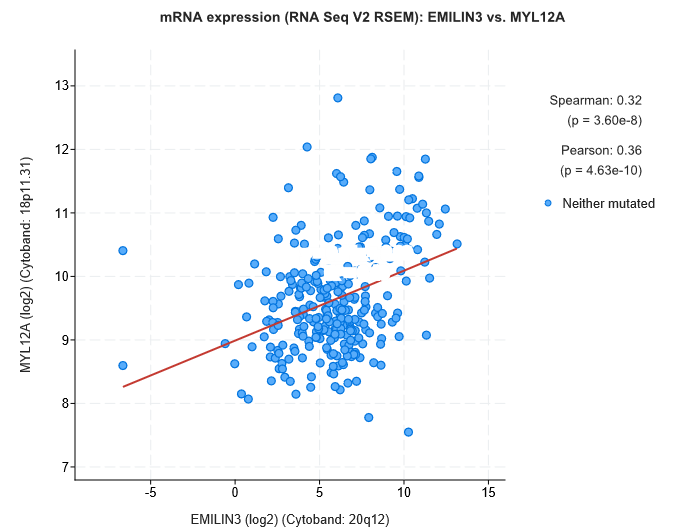

Supplement: Supplemental Information 1 [file peerj-08-8696-s001.zip › Raw data/co-expressed genes/MYL12A ╝⌠╟╨/MYL12A%20EMILIN3.png]

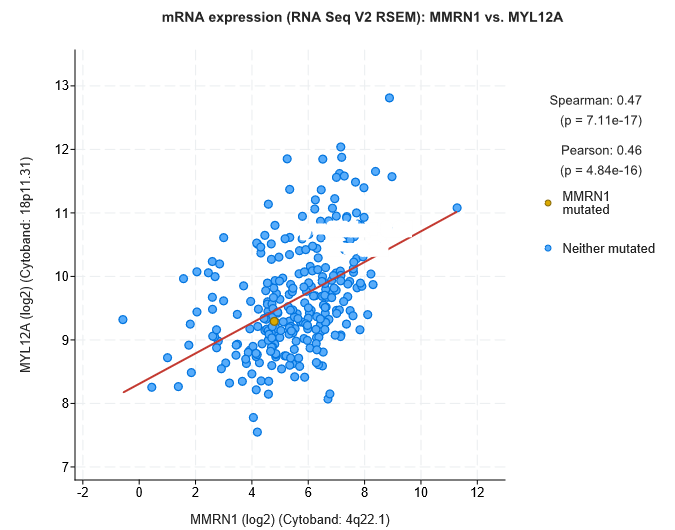

Supplement: Supplemental Information 1 [file peerj-08-8696-s001.zip › Raw data/co-expressed genes/MYL12A ╝⌠╟╨/MYL12A%20MMRN1.png]

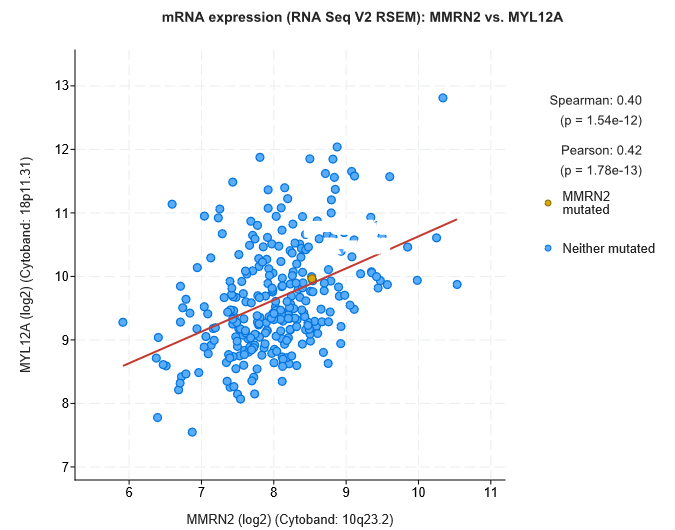

Supplement: Supplemental Information 1 [file peerj-08-8696-s001.zip › Raw data/co-expressed genes/MYL12A ╝⌠╟╨/MYL12A%20MMRN2.png]

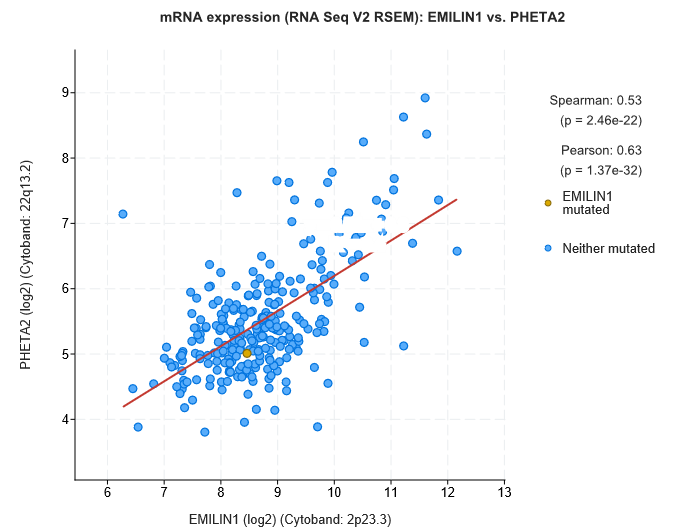

Supplement: Supplemental Information 1 [file peerj-08-8696-s001.zip › Raw data/co-expressed genes/PHETA2 ╝⌠╟╨/PHETA2%20EMILIN1.png]

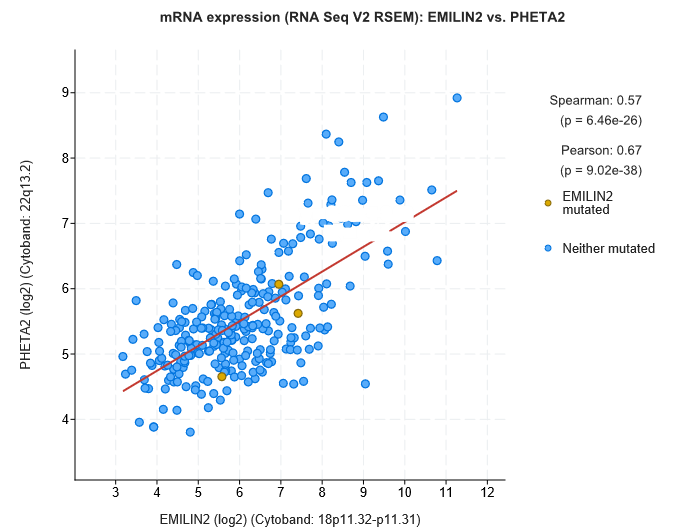

Supplement: Supplemental Information 1 [file peerj-08-8696-s001.zip › Raw data/co-expressed genes/PHETA2 ╝⌠╟╨/PHETA2%20EMILIN2.png]

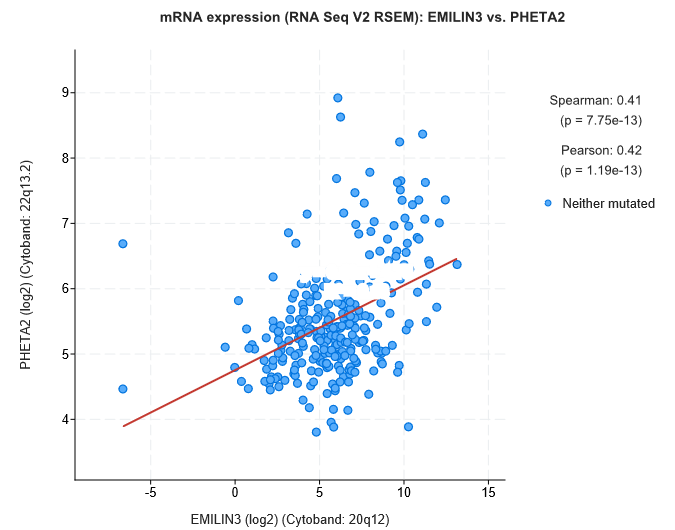

Supplement: Supplemental Information 1 [file peerj-08-8696-s001.zip › Raw data/co-expressed genes/PHETA2 ╝⌠╟╨/PHETA2%20EMILIN3.png]

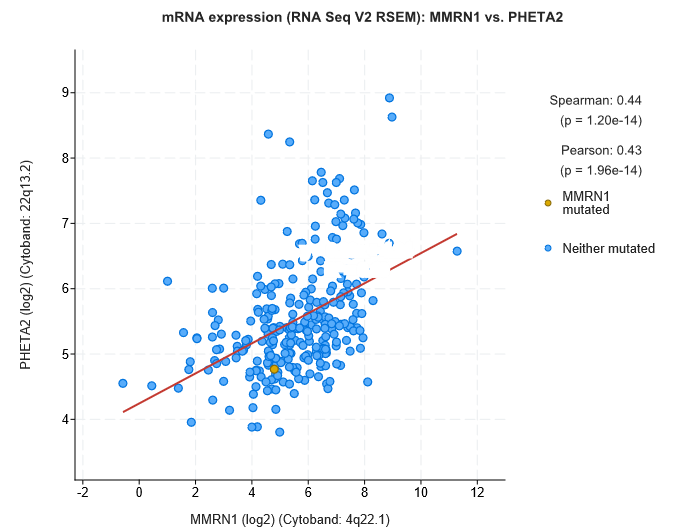

Supplement: Supplemental Information 1 [file peerj-08-8696-s001.zip › Raw data/co-expressed genes/PHETA2 ╝⌠╟╨/PHETA2%20MMRN1.png]

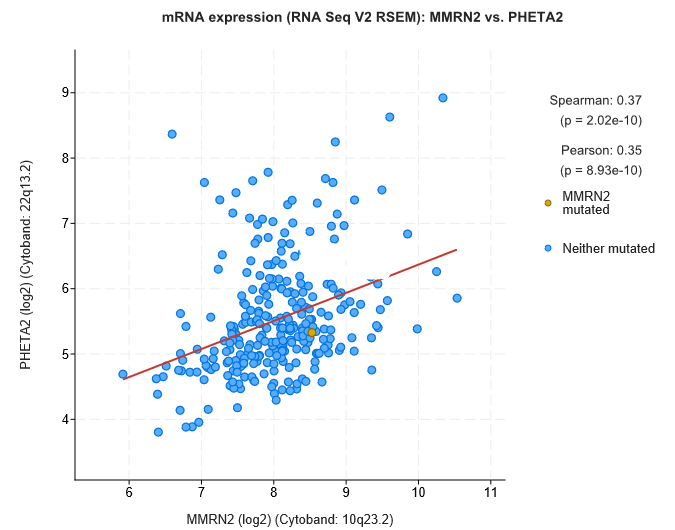

Supplement: Supplemental Information 1 [file peerj-08-8696-s001.zip › Raw data/co-expressed genes/PHETA2 ╝⌠╟╨/PHETA2%20MMRN2.png]

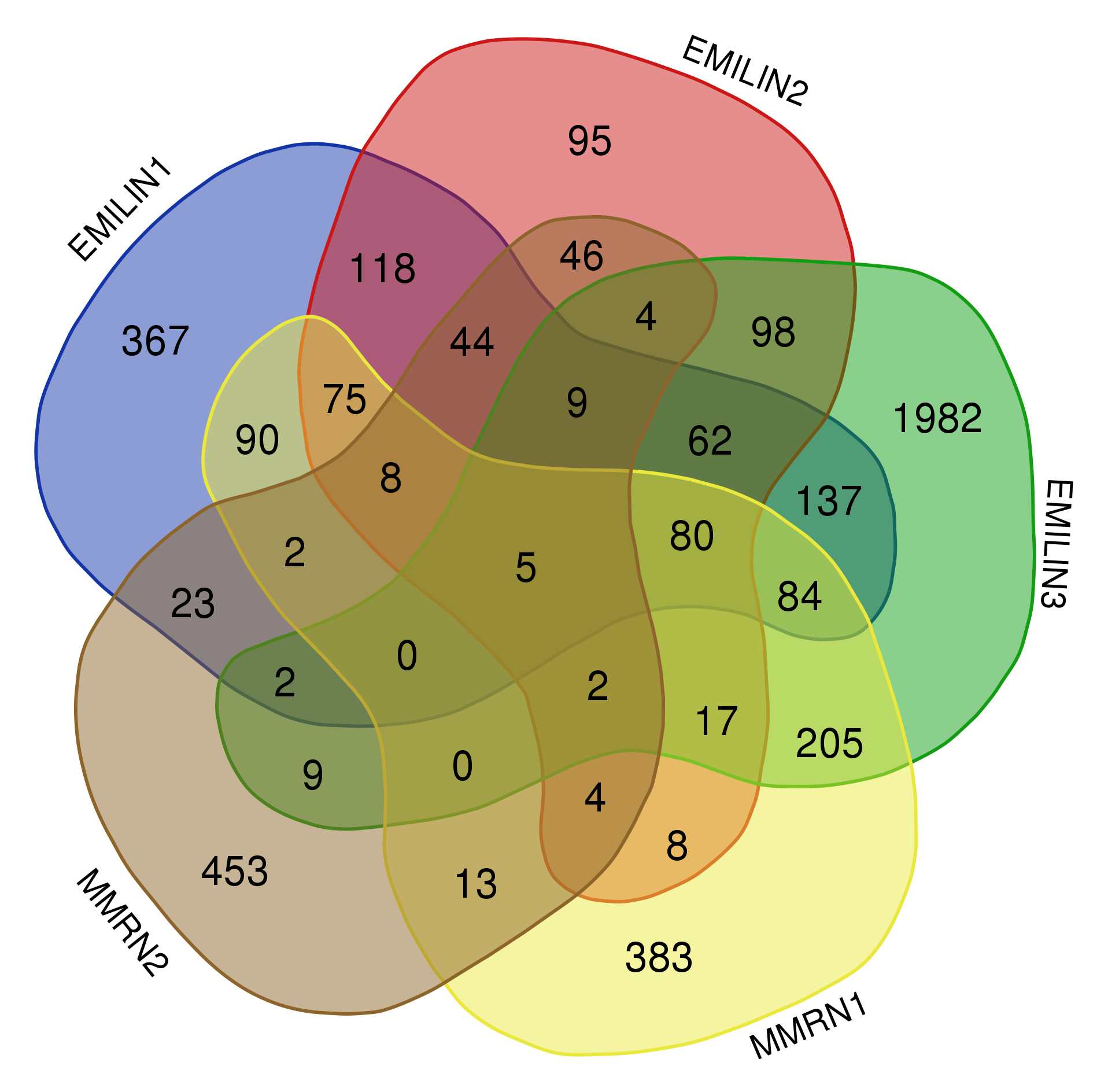

Supplement: Supplemental Information 1 [file peerj-08-8696-s001.zip › Raw data/co-expressed genes/venn_result3069.png]

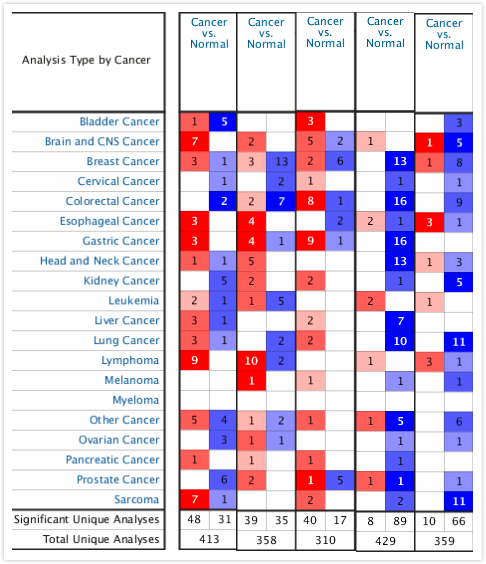

Supplement: Supplemental Information 1 [file peerj-08-8696-s001.zip › Raw data/oncomine/oncomine ╫≈═╝/ALL ║╧│╔║≤.jpg]

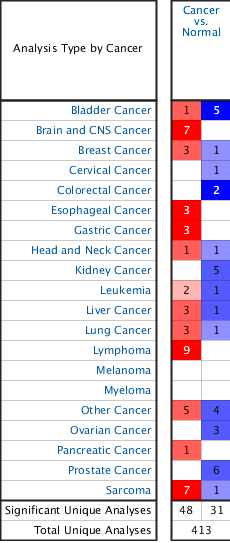

Supplement: Supplemental Information 1 [file peerj-08-8696-s001.zip › Raw data/oncomine/oncomine ╫≈═╝/EMILIN1 ALL.png]

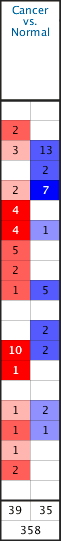

Supplement: Supplemental Information 1 [file peerj-08-8696-s001.zip › Raw data/oncomine/oncomine ╫≈═╝/EMILIN2 ALL.png]

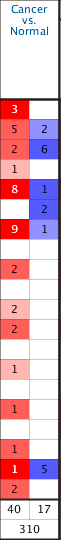

Supplement: Supplemental Information 1 [file peerj-08-8696-s001.zip › Raw data/oncomine/oncomine ╫≈═╝/EMILIN3 ALL.png]

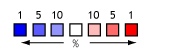

Supplement: Supplemental Information 1 [file peerj-08-8696-s001.zip › Raw data/oncomine/oncomine ╫≈═╝/FCH)8{[K5U0M2IFO`SCUGC0.png]

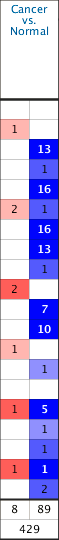

Supplement: Supplemental Information 1 [file peerj-08-8696-s001.zip › Raw data/oncomine/oncomine ╫≈═╝/MMRN1 ALL.png]

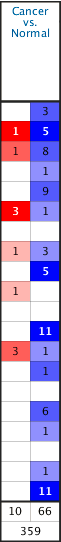

Supplement: Supplemental Information 1 [file peerj-08-8696-s001.zip › Raw data/oncomine/oncomine ╫≈═╝/MMRN2 ALL.png]

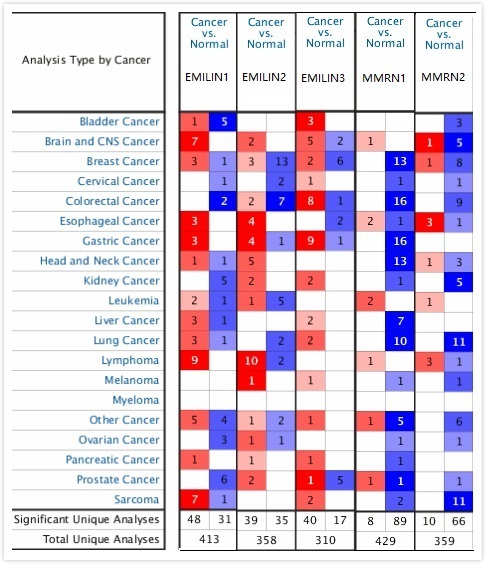

Supplement: Supplemental Information 1 [file peerj-08-8696-s001.zip › Raw data/oncomine/oncomine ╫≈═╝/┐╔╩╣╙├1.jpg]

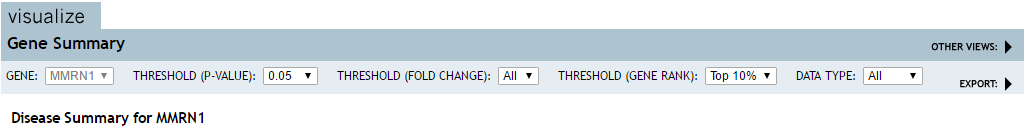

Supplement: Supplemental Information 1 [file peerj-08-8696-s001.zip › Raw data/oncomine/oncomine ╫≈═╝/╔╕╤í╠⌡╝■.png]
